# Risk Factors for Palbociclib-Induced Early Developing Neutropenia in Patients with Hormone Receptor-Positive Metastatic Breast Cancer

**DOI:** 10.3390/cancers15102810

**Published:** 2023-05-18

**Authors:** Yeonhong Lee, Dayae Lee, Inyoung Seo, Heejung Chae, Sung Hoon Sim, Keun Seok Lee, Hye Sun Gwak

**Affiliations:** 1 College of Pharmacy and Graduate School of Pharmaceutical Sciences, Ewha Womans University, Seoul 03760, Republic of Korea; yhlee@ncc.re.kr; 2 Department of Pharmacy, National Cancer Center, Goyang-si 10408, Republic of Korea; 13668@ncc.re.kr (D.L.); seoiy@ncc.re.kr (I.S.); 3 Center for Breast Cancer, National Cancer Center, Goyang-si 10408, Republic of Korea; hchae21@ncc.re.kr (H.C.); simsh@ncc.re.kr (S.H.S.)

**Keywords:** metastatic breast cancer, palbociclib, neutropenia, CDK4/6 inhibitor

## Abstract

**Simple Summary:**

Palbociclib, an oral cyclin-dependent kinase 4/6 inhibitor, in combination with endocrine therapy improved outcomes in the metastatic breast cancer patient. Neutropenia (NP) is the most common adverse event, with palbociclib being more frequent among Asians. To date, there is limited data on the factors that increase the risk of early-onset neutropenia after palbociclib treatment and whether neutropenia affects treatment outcomes. We conducted a retrospective study to investigate the risk factors associated with early-developing neutropenia in patients with HR+/HER2−metastatic breast cancer and evaluate median progression-free survival (PFS). Our study showed that early-developing NP was significantly associated with low baseline BSA, ANC, WBC, and PLT, and baseline ANC < 3700/mm^3^, WBC < 6.30 × 10^9^/mm^3^, PLT < 230 × 10^9^/mm^3^, and BSA < 1.58 m^2^ increased the risk by approximately 4.0-fold, 3.7–4.0-fold, 2.1-fold, and 2.0-fold, respectively. The occurrence of early-onset neutropenia did not affect median PFS (*p* = 0.710), although patients with neutropenia had more frequent dose reductions or treatment delays.

**Abstract:**

Purpose: This study aimed to determine the risk factors for palbociclib-induced grade 4 or grade 3 neutropenia (NP) requiring dose reduction or delayed treatment in patients with HR+/HER2−metastatic breast cancer in the first 3 cycles (early grade 3/4 NP) and whether the early developing grade 3/4 NP affects progression-free survival. Methods: A retrospective study using electronic medical records was conducted on patients who received palbociclib for metastatic breast cancer between January 2018 and August 2022. The early grade 3/4 NP risk factors were evaluated with univariate and multivariable logistic regression analyses. In addition, the Kaplan-Meier method was used to estimate the median progression-free survival (PFS) to analyze the effect of early grade 3/4 NP on treatment. Results: Out of the 264 patients included in this study, 173 (65.6%) experienced early grade 3/4 NP. A total of four models were applied for multivariable analysis to identify early grade 3/4 NP-developing factors. Low baseline ANC, WBC, PLT, and BSA were significant risk factors for early grade 3/4 NP; baseline ANC < 3700/mm^3^, WBC < 6.30 × 10^9^/mm^3^, PLT < 230 × 10^9^/mm^3^, and BSA < 1.58 m^2^ increased the risk by approximately 4.0-fold, 3.7–4.0-fold, 2.1-fold, and 2.0-fold, respectively. Early grade 3/4 NP did not affect PFS (*p* = 0.710), although patients with early grade 3/4 NP had more frequent dose reductions or treatment delays. Conclusions: Based on the results, low baseline ANC, WBC, PLT, and BSA were associated with early grade 3/4 NP. Patients with risk factors require careful monitoring, and this study is expected to help predict NP, which may appear in early treatment.

## 1. Introduction

Breast cancer is the most common cancer in women worldwide [1], while therapeutic options for patients with breast cancer are varied and complex according to molecular subtype [2]. Approximately 60–65% of breast cancer cases are hormone receptor-positive (HR+) and human epidermal growth factor 2-negative (HER2−) [3]. Endocrine therapy, targeting the estrogen signaling pathway, has been used as standard therapy for HR+/HER2− breast cancer. Aromatase inhibitors (AIs; e.g., letrozole, anastrozole, and exemestane), selective estrogen receptor modulators (e.g., fulvestrant), and selective estrogen receptor modulators (e.g., tamoxifen) have essential roles in the treatment of HR+/HER2− breast cancer. However, acquired resistance occurred in most patients; therefore, new approaches for novel targets to overcome the resistance are needed [4,5].

Cyclin-dependent kinases (CDKs) are a family of serine/threonine kinases that have an important role in the regulation of cell cycle progression [6]. CDK4/6 inhibitors such as palbociclib, ribociclib, and abemaciclib are reversible small molecular inhibitors of CDK 4 and 6. Through blocking cyclin D and CDK4/6, CDK4/6 inhibitors inhibit the phosphorylation of retinoblastoma and cell proliferation, thereby preventing cell cycle progression from G1 to S phase [7]. CDK4/6 inhibitors have rapidly changed the treatment landscape for ER+/HER2− metastatic breast cancer patients in combination with endocrine therapy, which significantly lengthened progression-free survival (PFS) and overall survival (OS) [8].

Palbiciclib was the first approved CDK4/6 inhibitor by the U.S. Food and Drug Administration in 2015 in patients with HR+/HER2− metastatic breast cancer. The previous phase Ⅲ clinical trials, PALOMA-2 and PALOMA-3, demonstrated that palbociclib prolonged PFS and OS in combination with endocrine therapy, letrozole in first-line ER+/HER2− advanced breast cancer patients [9], or fulvestrant in HR+/HER2− metastatic breast cancer patients with disease progression after previous endocrine therapy as second- or later-line treatment [10]. However, adverse events (AEs) were more frequent in the palbociclib-endocrine therapy group than in the placebo-endocrine therapy group. The most common AE was myelosuppression, particularly neutropenia (NP). The incidence rate of NP in all grades was observed in 80% of cases, with grade 3/4 accounting for 66% [9,11]. In addition, subgroup analysis of PALOMA-2 with the Asian population showed that hematologic toxicities were more frequent among Asian patients (all grades: 95.4%, grade 3/4: 89.2%) [12] compared to patients of other ethnicities.

Despite the high incidence of NP, it is reported that relatively few patients developed febrile NP, and the rate of permanent treatment discontinuation due to NP was low. The mechanism of NP associated with palbociclib is distinct from cytotoxic drug-induced NP [13] since palbociclib-induced myelosuppression involves the differentiation of precursor cells in the bone marrow through cell cycle arrest without DNA damage or apoptosis [14].

Palbociclib-induced NP is rapidly reversible when palbociclib is discontinued. Hence, an intermittent dosing schedule of 3-weeks on and 1-week off regimen is recommended to manage hematologic toxicity [15]. The onset of palbociclib-induced NP occurred predominantly during the first two to three weeks and lasted for an average of one week. Therefore, monitoring of the complete blood count on days 1 and 15 in the first two cycles is recommended, and dose reduction is recommended for grade 4 NP or grade 3 NP with febrile NP [16].

Palbociclib is well tolerated, and NP is manageable through dose reduction or treatment adjustment without affecting efficacy [17]. However, an increased incidence of NP may have negative effects on patients and ultimately reduce their quality of life. The incidence of NP has been reported to occur most frequently within the first 2 months of palbociclib treatment [18]. Therefore, risk factors that may predict palbociclib-induced grade 4 or grade 3 NP requiring dose reduction or delayed treatment in the first 3 cycles [19] (early grade 3/4 NP) could be helpful for the treatment, but there is limited data available. This study aimed to evaluate risk factors associated with palbociclib-induced early grade 3/4 NP and determine whether the occurrence of early grade 3/4 NP in patients with HR+/HER2− metastatic breast cancer affects treatment outcomes.

## 2. Methods

### 2.1. Patients and Study Design

A retrospective study was conducted at the National Cancer Center (Gyeonggi-do, Republic of Korea) in patients with HR+ and HER2−metastatic breast cancer who received palbociclib between January 2018 and August 2022. Eligibility criteria were patients with age ≥18 years who completed at least 3 cycles of palbociclib therapy or who discontinued treatment within 3 cycles due to hematological AEs. This study was approved by the Institutional Review Board (IRB) of the National Cancer Center in Korea (IRB number: NCC 2022-0309), and informed consent was waived due to the nature of a retrospective study.

Patients were treated with palbociclib plus endocrine therapy with either AI or fulvestrant. Patients received palbociclib once daily on a schedule of 3 weeks followed by 1 week off, administered orally. When AI was used as an endocrine partner, either letrozole 2.5 mg or exemestane 25 mg was administered orally once a day. Fulvestrant at a dose of 500 mg was administered intramuscularly every 2 weeks for the first 3 doses and then every 4 weeks. Premenopausal patients received either bilateral oophorectomy or LHRH agonists before palbociclib treatment. Individual patient data were obtained from electronic medical records. Patient demographics, including age, body weight, body surface area (BSA), body mass index (BMI), Eastern Cooperative Oncology Group Performance Status (ECOG PS), disease status, metastatic disease sites, numbers of metastatic disease sites, menopausal status, bilateral salpingo-oophorectomy (BSO), treatment line, prior chemotherapy/endocrine/prior/radiotherapy for metastatic disease, laboratory test, combination endocrine therapy, comorbidity, concomitant drugs, initial palbociclib dose, the occurrence of NP, as well as the start and end date of treatment were collected.

### 2.2. Outcome Measurements

To identify the risk factor for early grade 3/4 NP, patients were divided into two groups: the early grade 3/4 NP group and the non-early grade 3/4 NP group. Grades 3 and 4 NP were defined as absolute neutrophil counts <1000/m^3^ and <500/m^3^, respectively, according to the National Cancer Institute Common Terminology Criteria for Adverse Events (CTCAE), version 5.0. PFS was defined as the time from palbociclib initiation to confirmed disease progression or death. Disease progression was determined according to response evaluation criteria in solid tumors (RECIST) version 1.1.

### 2.3. Statistical Analysis

The chi-squared test and Fisher’s exact test were used for the analysis of qualitative variables, and the independent t-test was used for quantitative variables. A multivariable logistic regression model was performed to identify independent risk factors for early grade 3/4 NP after adjusting confounders (age and variables with *p* values below 0.05 in univariate analysis). To determine the optimal cutoff values for the prediction of risk factors, receiver operating characteristic (ROC) curve analyses were used, and independent risk factors were analyzed for multicollinearity (|r| > 0.7). Variables were entered by stepwise selection when *p* was lower than 0.05 and were removed when *p* was higher than 0.1. The unadjusted odds ratio (OR) and adjusted OR, with the 95% confidence interval (CI), were calculated from univariate and multivariate analyses, respectively. The Hosmer-Lemeshow goodness-of-fit test was performed to check the prediction’s fit. PFS was estimated with Kaplan–Meier methods and compared with the log-rank test. Two-sided *p*-values less than 0.05 were considered statistically significant, and all statistical analyses were conducted using the IBM SPSS Statistics for Windows, version 20.0 (IBM Corp., Armonk, NY, USA).

### 2.4. Results

Between January 2018 and August 2022, data from 264 metastatic breast cancer patients treated with palbociclib were included in the analysis, and 173 patients (65.5%) experienced early grade 3/4 NP.

Baseline patient characteristics are summarized in Table 1. All patients were female, and the median age was 55 years (range 29–90 years) in the early grade 3/4 NP group and 56 years (range 35–90 years) in the non-early grade 3/4 NP group. Overall, most patients had ECOG PS 0–1, and 7.6% of patients showed ECOG PS 2–3. Sixty-eight patients (25.8%) were diagnosed with de novo metastatic breast cancer, and the site of metastatic disease was visceral in 58.7% of patients and nonvisceral in 41.3%. Most patients were postmenopausal, and 28.2% of premenopausal patients received bilateral salpingo-oophorectomy (BSO) within a month before starting palbociclib for therapeutic purposes. Approximately 10–20% of patients received prior chemotherapy, endocrine therapy, or radiotherapy for metastatic disease, and 76.9% of patients received palbociclib as first-line therapy. The initial dose of palbociclib was 125 mg in 87.1% of patients, and the majority of patients (79.5%) used aromatase inhibitors as a part of their combined endocrine therapy, and 20.5% used fulvestrant. BSA, BMI, and baseline laboratory tests, including absolute neutrophil (ANC), white blood cell (WBC), hemoglobin (Hb), and platelet (PLT), showed significant differences between the early grade 3/4 NP group and the non-early grade 3/4 NP group.

Table 2 shows the result of univariate and multivariable logistic regression analyses regarding risk factors for early grade 3/4 NP after palbociclib treatment, using variables with *p*-values less than 0.05 in addition to age. Using the ROC analyses, the optimal cut-off values for ANC, WBC, PLT, BSA, and BMI were calculated, which were 3700/mm^3^ (AUC = 0.718), 6.30 × 10^3^/mm^3^ (AUC = 0.732), 230 × 10^3^/mm^3^ (AUC = 0.627), 1.58 m^2^ (AUC = 0.571), and 23 kg/m^2^ (AUC = 0.564), respectively. In the univariate analysis, BSA < 1.58 m^2^, BMI < 23 kg/m^2^, ANC < 3700/mm^3^, WBC < 6.30 × 10^3^/mm^3^, and PLT < 230 × 10^3^/mm^3^ were significant risk factors for early grade 3/4 NP. 

Since there were collinearities between BMI and BSA (r = 0.712) as well as between ANC and WBC (r = 0.903), four models were constructed for multivariable analysis. Model Ⅰ included age, BSA, ANC, and PLT. Model Ⅱ included WBC instead of ANC of model Ⅰ. Models Ⅲ and Ⅳ included BMI instead of BSA. Along with other risk factors, ANC and WBC were included in models Ⅲ and Ⅳ, respectively. 

**Figure 1:**
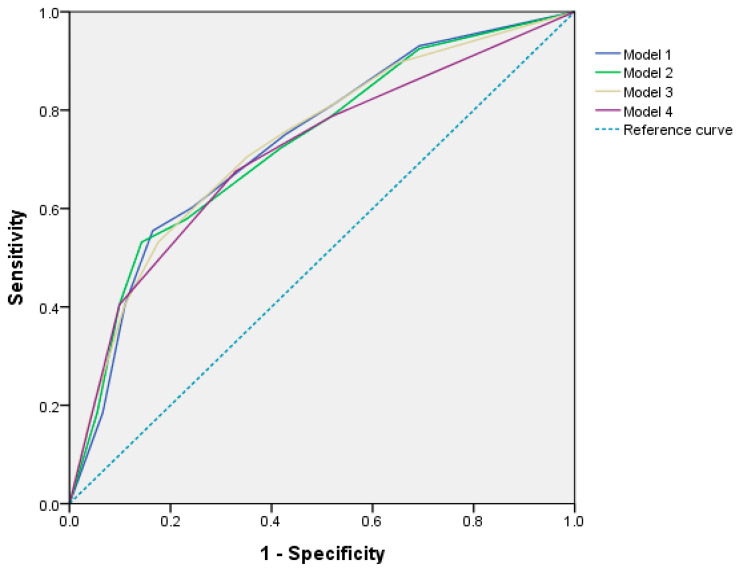
The receiver operating characteristic (ROC) curve for the predictive performance of four models from multivariable logistic regression.

Based on models Ⅰ and Ⅲ, ANC < 3700/mm^3^ increased the incidence of early grade 3/4 NP by approximately 4.0–4.1-fold. In models Ⅱ and Ⅳ, WBC < 6.30 × 10^3^/mm^3^ increased the incidence of early grade 3/4 NP by 3.7–4.0-fold. BSA < 1.58 m^2^ was a risk factor for early grade 3/4 NP, which increased 1.9–2.0-fold in models Ⅰ and Ⅱ; however, the BMI did not show the significance for the incidence of early grade 3/4 NP from the multivariable analysis. PLT < 230 × 10^3^/mm^3^ was a factor that increased the risk of early grade 3/4 NP by 2.1–2.2-fold in all models. The Hosmer–Lemeshow test for NP revealed a good fit for the four models (*p* = 0.307, 0.217, 0.910, and 0.296, respectively). AUROC values from multivariable logistic regression indicated the acceptable performance of the models Ⅰ, Ⅱ, III, and Ⅳ, which were 0.735 (95% CI 0.671–0.798), 0.730 (95% CI 0.667–0.793), 0.734 (95% CI 0.671–0.797), and 0.713 (95% CI 0.650–0.777), respectively (Figure 1).

**Figure 2:**
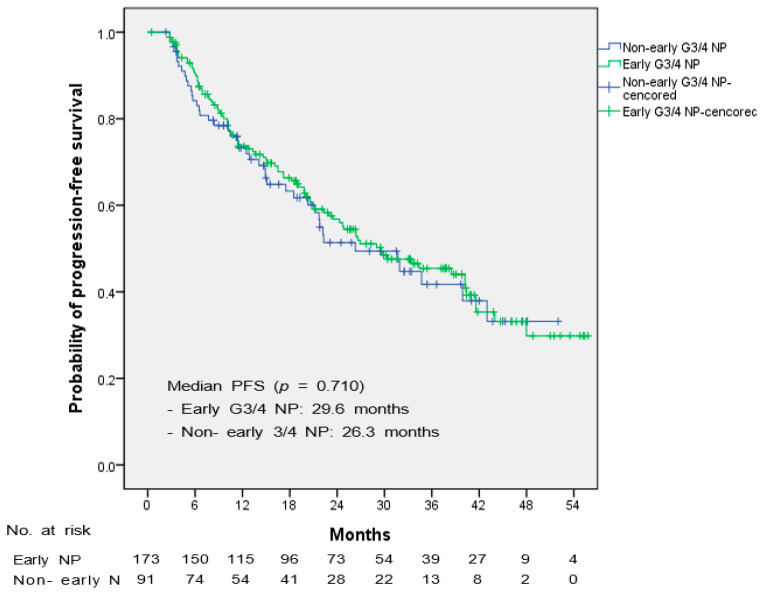
Kaplan-Meier curve showing the difference in progression-free survival between the early grade 3/4 NP group and the non-early grade 3/4 group.

The median PFS was evaluated to determine whether the occurrence of early NP affected the treatment effect, and the Kaplan–Meier survival curve is shown in Figure 2. At the time of the data cutoff (August 2022), 142 patients (49.6%) had disease progression. The median PFS was 29.0 months (95% CI 22.00–35.99). Among these patients, there was no difference in median PFS between patients with and without early grade 3/4 NP (*p* = 0.710). The median PFS of the early grade 3/4 NP group and non-early grade 3/4 NP group were 29.6 months (95% CI 19.43–39.76) and 26.3 months (95% CI 16.11–36.49), respectively. The PFS rates at 6 months and 12 months of the early grade 3/4 NP group and non-early grade 3/4 group were 90.5% and 84.2%, and 73.7% and 73.3%, respectively. 

## 3. Discussion

This study evaluated the risk factors affecting the development of early grade 3/4 NP in patients with HR+/HER2− metastatic breast cancer. BSA, baseline WBC, ANC, and PLT were significant risk factors for early grade 3/4 NP.

In this study, BSA < 1.58 m^2^ was associated with an increased risk of early grade 3/4 NP, resulting in an approximately 2.0-fold increase in the incidence of early grade 3/4 NP. Since physical size is known to affect pharmacokinetics, most cytotoxic agents use BSA-based dosing to reduce inter-patient pharmacokinetic variability [20]. However, targeted oral therapy, including CDK4/6 inhibitors and tyrosine kinase inhibitors (TKIs), uses a fixed dose regardless of weight or BSA. It has been shown that there is an exposure-efficacy/toxicity outcome relationship between palbociclib [21,22] and TKIs [23,24]. Leenhardt et al. showed that the plasma C_trough_ of palbociclib has a correlation with developing high-grade NP. Roncato et al. showed that the minimum plasma concentration of CDK4/6 inhibitors above the mean value caused hematologic AE, including NP. Larson et al. showed imatinib had an inverse correlation between BSA and blood concentration [25]. These results indicate a correlation between blood concentration and BSA that supports the role of BSA as a risk factor for early grade 3/4 NP in patients with palbociclib, where NP occurs more frequently in patients with low BSA.

Clinical trial data has shown that patients with a lower baseline ANC were associated with increased NP risk and that Asian patients with more NP had 18–20% lower baseline ANC compared to non-Asian patients [12,26]. In the present study, baseline ANC was about 30% lower in patients with early grade 3/4 NP than in those without it. This study constructed four models to evaluate risk factors for early grade 3/4 NP and found baseline ANC < 3700/mm3, the optimal cutoff value from the ROC curve, as a significant risk factor. Similarly, Kimura et al. [27] and Vazquez et al. [19] suggested a predictive value for severe NP as an ANC lower than 3680/mm^3^ and 3370/mm^3^, respectively.

Other than ANC, this study also suggested WBC < 6.30 × 10^3^/mm^3^ and PLT < 230 × 10^3^/mm^3^ as significant risk factors for early grade 3/4 NP. Lavery et al. [28] indicated that baseline myelosuppression, including low ANC and PLT, is a risk factor for grade 3/4 neutropenia. Iwata et al. [29] showed that the low baseline WBC and PLT were significant variables for early-developing grade 3/4 neutropenia from univariate analysis. However, multivariable analysis could not be performed due to multicollinearity issues among variables. Kanbayashi et al. [30] suggested low baseline PLT as a risk factor, although it was not significant because of the small sample size. Since data related to WBC and PLT on the early occurrence of grade ¾, neutropenia is still limited, more research is needed.

There was no risk factor found in comorbidity and concomitant drugs. Palbociclib is a weak CY3A4 inhibitor and major substrate [31] that may have a drug interaction between a CYP3A4 inhibitor and an inducer. In this study, the CYP3A4 inhibitor did not indicate a significant risk factor for early grade 3/4 NP, which corresponds with earlier study results that CYP3A4 inhibitors did not significantly affect the incidence of high-grade NP [21]. However, inter-individual variability of CDK4/6 inhibitors due to pharmacokinetic variables, including drug-drug interactions and drug-genetic interactions, has been reported that may alter the pharmacokinetic profile and plasma concentration of palbociclib. Close monitoring is necessary when the concomitant administration of palbociclib with drugs may affect drug absorption, distribution, metabolism, and elimination (ADME) and the expression of relevant ADME genes (e.g., CYP3A4, CYP3A5, or ABGB1) [22].

Since palbociclib is a weak base drug with a pH-dependent solubility, coadministration of a proton pump inhibitor or H2 blocker may alter the gastrointestinal tract pH, influencing its absorption [32,33,34]. However, no statistical significance was found in the present study. In addition, there was no difference between patients with early grade 3/4 NP and without early grade 3/4 NP regarding age, metastatic site, prior chemotherapy, endocrine therapy, or radiotherapy.

The median PFS was not significantly different between early grade 3/4 NP and non-early grade 3/4 groups. Although the early grade 3/4 NP group had more frequent dose reduction or treatment delay, the PFS rate at 6 months was higher in the early grade 3/4 NP group compared to the non-early grade 3/4 group, which was 90.5% and 84.2%, respectively. From these findings, it is inferred that dose reduction or delay due to early-developing grade 3/4 NP does not adversely affect efficacy, and several previous studies indicated consistent results [12,35,36].

It is controversial whether the occurrence of grade 3/4 NP affects median PFS. McAndrew et al. reported a strong association between the early onset of NP and PFS [37]; however, there was no difference in PFS among patients who experienced grade 3/4 NP versus grade 2 or less in the PALOMA-3 study [9]. Our study results also indicated that there was no statistical difference. However, more studies are needed to confirm these results.

Several limitations should be considered in this study. It was a retrospective study, so some data were missing or underestimated. Although analysis of comorbidity and concomitant medications was included in this study, sufficient data could not be obtained (from data on comorbidity and concomitant drugs managed at a local hospital). Nevertheless, this study provides real-world data demonstrating risk factors associated with early grade 3/4 NP in the Asian population with a relatively large number of patients. Further studies are required to confirm these findings.

## 4. Conclusions

In conclusion, our study demonstrated that BSA and baseline myelosuppression, including WBC, ANC, and PLT, were associated with palbociclib-induced early grade 3/4 NP in patients with HR+/HER2− to metastatic breast cancer. The occurrence of grade 3/4 NP, which led to more frequent dose reductions or delays in treatment, did not appear to affect the efficacy of palbociclib. Nevertheless, these results should be confirmed with prospective multicenter studies.

## Figures and Tables

**Table 1:** Patient demographic and baseline characteristics.

Characteristic	Total	Early G3/4 NP	Non-Early G3/4 NP	*p*
	(n = 264)	(n = 173)	(n = 91)	
Age, years—median (range)				
	55 (29–90)	55 (29–90)	56 (35–90)	0.751
Weight (kg)—n (%)				
<58	130 (49.2)	92 (53.2)	38 (41.8)	0.078
≥58	134 (50.8)	81 (46.8)	53 (58.2)	
BSA (m^2^)—n (%)				
<1.58	119 (45.1)	86 (49.9)	33 (36.3)	0.037
≥1.58	145 (54.9)	87 (50.3)	58 (63.7)	
BMI (kg/m^2^)—n (%)				
<23	105 (39.8)	77 (45.8)	28 (30.8)	0.030
≥23	159 (60.2)	96 (55.5)	64 (69.2)	
ECOG PS—n (%)				0.382
0	98 (37.3)	64 (37.2)	34 (37.4)	
1	98 (37.3)	69 (40.1)	29 (31.9)	
≥2	20 (7.6)	11 (6.4)	9 (9.9)	
Unknown	47 (17.9)	28 (16.3)	19 (20.9)	
Disease status—n (%)				0.896
De novo	68 (25.8)	45 (26.0)	23 (25.3)	
Recurrence	196 (74.2)	128 (74.0)	68 (74.7)	
Metastatic disease site—n (%)				0.367
Viceral	155 (58.7)	105 (60.7)	50 (54.9)	
Nonviceral	109 (41.3)	68 (39.3)	41 (45.1)	
Bone	154 (58.3)	94 (54.3)	60 (65.9)	0.069
Bone only	53 (20.1)	32 (18.5)	21 (23.1)	0.377
Metastatic disease site—n (%)				0.211
1	119 (45.1)	80 (46.2)	39 (42.9)	
2	83 (31.4)	58 (33.5)	25 (27.5)	
≥3	62 (23.5)	35 (20.2)	27 (29.7)	
Menopausal status—n (%)				0.739
Premenopausal	7 (2.7)	5 (2.9)	2 (2.2)	
Postmenopausal	257 (97.3)	168 (97.1)	89 (97.8)	
BSO in a Month—n (%)				0.818
Yes	76 (28.2)	49 (28.3)	27 (29.7)	
No	188 (71.1)	124 (71.7)	64 (70.3)	
Treatment line—n (%)				0.939
1	203 (76.9)	134 (77.5)	69 (75.8)	
2	24 (9.1)	15 (8.7)	9 (9.9)	
≥3	37 (14.0)	24 (13.9)	13 (35.1)	
Prior Chemotherapy for metastatic disease—n (%)				0.807
Yes	33 (12.5)	21 (12.1)	12 (13.2)	
No	231 (87.5)	152 (87.9)	79 (86.8)	
Prior Endocrine therapy for metastatic disease—n (%)				0.499
Yes	52 (19.7)	32 (18.5)	20 (22.0)	
No	212 (80.3)	141 (81.5)	71 (78.0)	
Prior radiotherapy—n (%)				0.686
Yes	41 (15.5)	28 (16.2)	13 (14.3)	
No	223 (84.5)	145 (83.8)	78 (85.7)	
Bone RT	31 (11.7)	24 (13.9)	7 (7.7)	0.138
Bone RT in a year	21 (8.0)	17 (9.8)	4 (4.4)	0.121
Baseline laboratory test—median (IQR)				
ANC (cell/mm^3^)	3401 (2468–4567)	3072 (2273–5081)	4356 (3130–5304)	<0.001
WBC (×10^3^/mm^3^)	6.04 (4.76–7.42)	5.44 (4.26–6.65)	6.87 (5.99–8.67)	<0.001
Hb (g/dL)	12.5 (11.5–13.5)	12.5 (11.6–13.5)	12.8 (11.8–14.7)	0.033
PLT (×10^3^/mm^3^)	236 (191–293)	227 (187–285)	258 (221–315)	0.001
Total bilirubin(mg/dL)	0.5 (0.4–0.6)	0.5 (0.4–0.7)	0.5 (0.4–0.6)	0.509
AST (IU/L)	23 (20–32.5)	24 (20–33)	22 (18–31)	0.822
ALT (IU/L)	18 (13–29)	19 (13–31)	17 (13–26)	0.879
Comorbidities—n (%)				
Hypertension	86 (32.6)	58 (33.5)	28 (30.8)	0.65
DM	50 (18.9)	28 (16.2)	22 (24.2)	0.115
Dyslipidemia	49 (18.6)	32 (18.5)	17 (18.7)	0.971
Concomitant medication—n (%)				
Denosumab	76 (28.8)	51 (29.5)	25 (27.5)	0.732
CaD	169 (64.0)	110 (63.6)	59 (64.8)	0.84
CYP3A4 inhibitor	47 (17.8)	31 (17.9)	16 (17.6)	0.946
ARB	55 (20.8)	39 (22.5)	16 (17.6)	0.346
Statin	63 (23.9)	41 (23.7)	22 (24.2)	0.931
H2 blocker	13 (4.9)	8 (4.6)	5 (5.5)	0.756
PPI	16 (6.1)	10 (5.8)	6 (6.6)	0.792
Antacid	18 (6.8)	13 (7.5)	5 (5.5)	0.536
Combination—n (%)				0.582
AI	210 (79.5)	140 (80.9)	70 (76.9)	
Fulvestrant	54 (20.5)	33 (19.1)	21 (23.1)	
Initial dose—n (%)				0.092
125 mg	230 (87.1)	156 (90.2)	74 (81.3)	
100 mg	30 (11.4)	15 (8.7)	15 (16.5)	
75 mg	4 (1.5)	2 (1.2)	2 (2.2)	

AI: aromatase inhibitor; ANC: absolute neutrophil count; ALT: alanine transaminase; ARBs: angiotensin II receptor blockers; AST: aspartate transaminase; BMI: body mass index; BSA: body surface area; BSO: bilateral salpingo-oophorectomy; CaD: calcium/cholecalciferol; CYP3A4: cytochrome P450 3A4, ECOG PS: Eastern Cooperative Oncology Group performance status; Hb: hemoglobin; NP: neutropenia; PLT: platelet; PPI: proton pump inhibitor; RT: radiotherapy; WBC: white blood cell. AI includes 209 letrozole and 1 exemestane.

**Table 2:** Univariate and multivariable regression analyses to identify predictors for early grade 3/4 neutropenia.

Predictors	Unadjusted OR	Model Ⅰ	Model Ⅱ	Model Ⅲ	Model Ⅳ
	(95% CIs)	Adjusted OR	Adjusted OR	Adjusted OR	Adjusted OR
		(95% CI)	(95% CI)	(95% CI)	(95% CI)
Age < 60 years	1.021 (0.600–1.739)				
BSA < 1.58 m^2^	1.737 (1.032–2.925) *	1.916 (1.087–3.376) *	2.050 (1.160–3.623) *		
BMI < 23 kg/m^2^	1.805 (1.055–3.087) *				
ANC < 3700/mm^3^	4.411 (2.569–7.571) ***	4.110 (2.350–7.189) ***		3.973 (2.281–6.919) ***	
WBC < 6.30 × 10^3^/mm^3^	4.248 (2.474–7.296) ***		4.043 (2.295–7.120) ***		3.732 (2.148–6.483) ***
PLT < 230 × 10^3^/mm^3^	2.649 (1.538–4.563) ***	2.194 (1.229–3.915) **	2.108 (1.180–3.766) *	2.083 (1.170–3.711) *	2.091 (1.180–3.707) *

ANC: absolute neutrophil count; BMI: body mass index; BSA: body surface area; OR: odds ratio; PLT: platelet; WBC: white blood cell. * *p* < 0.05, ** *p* < 0.01, *** *p* < 0.001. For Model Ⅰ construction, age, BSA, ANC, and PLT were included for analysis. For Model Ⅱ construction, age, BSA, WBC, and PLT were included for analysis. For Model Ⅲ construction, age, BMI, ANC, and PLT were included for analysis. For Model Ⅳ construction, age, BMI, WBC, and PLT were included for analysis.

## Data Availability

The datasets used in and/or analyzed during the current study are available from the corresponding author upon reasonable request.
